# Mental Health Assessments and Models of Care in the Emergency Department: A Narrative Review

**DOI:** 10.1111/1742-6723.70268

**Published:** 2026-04-27

**Authors:** Samantha E. Russell, Darlene L. Cole, Margaret M. Clarke, Michael Mennen, Stavroula Rebis, Lucy Horgan, Krishna Vakil, Jenny Choi

**Affiliations:** ^1^ Grampians Health Ballarat Victoria Australia; ^2^ Deakin Rural Health Deakin University Warrnambool Victoria Australia

**Keywords:** emergency department, mental health, model of assessment, model of intervention, models of care

## Abstract

Emergency departments are seeing an increase in mental health presentations across the globe; however, there are no standard mental health assessment models of care currently being used in emergency departments. This narrative review aims to search the literature and report the mental health models of care used globally. This narrative review conducted a search of 4 medical databases, Medline Complete, CINAHL, Embase and PsychINFO; the research team screened titles and abstracts and reviewed the full text. Risk of bias and quality assessment was conducted by 2 independent researchers. A total of 2461 articles were identified for title and abstract screening; of these, 84 were reviewed in full text and subsequently, 79 articles were excluded, resulting in 5 articles to be included in this narrative review. Two articles assessed the Psychiatric Assessment and Planning Unit (PAPU) model and reported on reduced length of stay; a third article reported a similar model and improved length of stay. Two articles reported models on sub‐sample cohorts of mental health: individuals experiencing suicide ideation and individuals experiencing alcohol and substance use issues. The results of this narrative review highlight the lack of literature concerning models of care and mental health assessment in emergency departments. Of the few articles reviewed, a successful model of care and/or assessment requires a dedicated physical space in the emergency department (such as a PAPU), including mental health short stay beds and dedicated mental health staff.

## 1 Introduction

Mental health disorders are debilitating and can place a large burden on an individual's life, and seeking support can be challenging. Mental health and substance use disorders were accounting for 15% of the total burden disease as measured by disability‐adjusted life years, an increase of 31% between the years 2003 and 2023, this comes a close second to cancer at 17% according to reports from the Australian Burden of Disease Study 2023 [1]. Approximately half of the world's population will develop one or more mental disorders by the age of 75 [2] and approximately 15% of Australians experienced high to very high levels of psychological distress in 2023–2024. Which is a marked increase from 2011 to 2012 data where 4% of males and 6% of females experienced psychological distress [3]. Additionally, studies on psychological distress report only minor differences in distress levels before COVID‐19 (Coronavirus) and during, indicating psychological distress is also prevalent within the population [4, 5].

In addition to the increasing reports of mental health disorders and psychological distress levels, there is an increase in people seeking help for mental health concerns. The 2020–2022 National Study of Mental Health and Wellbeing collected data on mental health service access in the preceding 12 months [2]. From this survey, it is estimated that 3.4 million Australians aged 16–85 saw a health professional for their mental health in the previous 12 months [2]. Hospital emergency departments (ED) are one of the places that individuals go to for mental health support, particularly in a regional or rural area where no other supports are available [6, 7, 8]. Mental health attendance rates to hospital emergency departments vary between an estimated 4.2% and 6.3% worldwide. Estimated prevalence rates for this cohort are challenging to measure as seen by the estimated prevalence gap [9, 10, 11]. Moreover, Australia's emergency department presentation for the year 2024–2025 was reported as 9.1 million [12]. Despite the large numbers of the population seeking mental health supports from emergency departments, there is currently no standardised model of assessment for mental health within hospital EDs. The literature on this topic states there are multiple models of mental health assessment [13, 14, 15, 16, 17] across the world and with differing age ranges, each with their own benefits and limitations, but no gold standard recommended model. However, there are numerous models of care for physical health concerns in EDs, including patient journey mapping, and individually described models from triage to discharge [18, 19, 20]. These models have been well established and implemented for decades with ongoing improvements, but without any considerations for mental health presentations in ED. The lack of well‐defined standards calls for work to be done to develop and implement new models of care for individuals with mental health concerns. This narrative review conducted by a team of clinicians and researchers at an Australian regional health service aims to identify the current models of care and/or assessment for emergency departments concerning mental health concerns.

## 2 Methods

We conducted a narrative review from database conception up to July 28, 2025, to understand the landscape of mental health assessment models in the emergency department. Specifically, our research question is: What are the mental health models of assessment and/or care for emergency departments? This review is structured according to the Preferred Reporting Items for Systematic Reviews and Meta‐analysis Extension for Scoping Reviews (PRISMA‐ScR) Guidelines [21]. See Supporting Information for PRISMA‐ScR checklist.

### 2.1 Information Sources and Databases

Relevant articles were identified through electronic searches of Medline Complete, EMBASE, CINAHL, PSYCHInfo. Databases were searched by S.E.R. from their inception to July 28, 2025.

### 2.2 Key Search Terms

We used the search framework Population, Concept and Context (PCC) to develop the appropriate search terms relating to the topic of interest. A total of 13 search terms were used in the search: 5 for the concept “mental health,” 4 for the concept “assessment” and 4 for the concept “emergency department” (e.g., Population: *mental health, mental disorder, psychiatric disorder, Concept: assessment, assessment model, screen, Context: emergency room, accident and emergency*). All search terms used the corresponding Boolean phrase limiters and MeSH (Medical Subject Headings) terms according to each of the 4 databases utilised. For example, see Appendix 1 for the search strategy utilised for the Medline Complete database.

### 2.3 Inclusion Criteria and Exclusion Criteria

We used the following inclusion criteria:

- Mental health patients, including substance abuse
- Crisis assessment/models
- All ages
- Emergency department/rooms

We used the following exclusion criteria:

- Physical health patients
- Patients in a community (outpatient), residential, or inpatient setting
- Non‐English articles
- Unpublished articles
- Protocols
- Reviews

### 2.4 Data Extraction

Six independent reviewers (D.C., M.M., L.H., S.R., M.C., S.E.R.) screened articles for eligibility. The abstract and title screening, full‐text screening, extraction of data, and assessment of bias were conducted by the research team (D.C., M.M., L.H., S.R., S.E.R., M.C., K.V. and J.C.). Author S.E.R. acted as adjudicator for to resolve any discrepancies at each stage. Studies were exported from the databases into Covidence systematic review software [22]. Variables collected from the studies were reported in the Summary of Findings Table 1. Which included: Author, study design, intervention, cohort, % of female participants, country of study, ages overall with mean and standard deviation, primary outcome of study and findings.

**TABLE 1:** Summary of findings.

Author, study design	Intervention	Cohort	% Female	Country	Age, overall mean (SD) y	Primary outcome	Findings
Browne et al. (2011), retrospective chart study	PAPU	Mental health	N/A	Australia	N/A	LOS	Study showed significant improvements in LOS in 12 months
Howard et al. (2019), feasibility study	AOD model screening pilot	AOD	40	Australia	45.7	Feasibility of model	Study showed model successful use in ED
Lester et al. (2018), retrospective chart review	CALM	Mental health	51	USA	31.4	LOS	Study showed significant improvements in LOS in 12 months
de Santiago‐Diaz et al. (2024), observational	CARS suicide reduction model	Suicidal	59	Spain	42.6	Suicidal behaviours and hospital readmission rates	Study showed significant reductions in suicidal behaviours and admissions at 12 month follow up
Mitchell et al. (2020), retrospective chart review and semi‐structured interviews (mixed methods)	PAPU	Mental health	N/A	Australia	N/A	LOS	Study showed significant reductions in LOS at one hospital but increased at the other 2 hospitals in evaluation

Abbreviations: AOD, alcohol and other drug; CALM, crisis assessment link and management; CARS, programme for management of suicidal behaviours and suicide prevention; ED, emergency department; LOS, length of stay; PAPU, psychiatric assessment and planning unit.

### 2.5 Data Analysis

Narrative assessment was conducted on the eligible articles. Each assessment model was reported in a summary of findings table and narratively described in the text.

### 2.6 Assessment of Risk of Bias and Quality Appraisal

Risk of bias for non‐randomised studies of Interventions was assessed using the RoBANS 2 [23] A Revised Risk of Bias Assessment Tool for Nonrandomised Studies of Interventions. Two independent reviewers (S.E.R. and K.V.) conducted all risk of bias assessments. Discrepancies between appraisals was resolved through discussion. For a summary of bias scores for each article, see Table 2. Quality appraisal was conducted utilising the Grading of Recommendation, Assessment, Development and Evaluation Assessment as Reported for Narrative Assessment (GRADE) assessment [24, 25] by authors S.E.R. and M.M.C. For summary scores for the certainty assessment for each article see Table 3.

**TABLE 2:** Revised version of the risk of bias tool for non‐randomised studies (RoBANS 2).

Risk of bias									
Article	Domain 1. comparability	Domain 2. selection	Domain 3. confounders	Domain 4. measurement	Domain 5. blinding	Domain 6. outcome assessment	Domain 7. incomplete data	Domain 8. selective reporting	Overall
Browne et al. (2011)	Unclear	Unclear	Low	High	Unclear	High	High	High	High risk
Howard et al. (2019)	Unclear	Unclear	High	High	Unclear	High	Unclear	Low	High risk
Lester et al. (2018)	Low risk	Low risk	Low risk	Low risk	High risk	Low risk	Unclear	High risk	Low risk
de Santiago Diaz et al. (2024)	Low risk	Low risk	Low risk	High risk	Low risk	High risk	Low risk	Low risk	Low risk
Mitchell et al. (2020)	Low risk	Low risk	Low risk	Low risk	High risk	Low risk	Low risk	Low risk	Low risk

**TABLE 3:** Grading of recommendation, assessment, development and evaluation assessment as reported for narrative assessment (GRADE).

Quality of evidence				
Article	Outcome	Participant no.	Effect	Quality of evidence (GRADE)
Browne et al. (2011)	PAPU reduced pre‐post LOS	N/A	Study showed significant improvements in LOS in 12 months	Moderate certainty due to high risk of bias
Howard et al. (2019)	AOD model screening pilot	*N* = 1100	Study showed model successful use in ED	Moderate certainty due to high risk of bias
Lester et al. (2018)	CALM model reduced pre‐post LOS	*N* = 2387	Study showed significant improvements in LOS in 12 months	Moderate certainty due to low risk of bias and moderate indirectness and imprecision
de Santiago Diaz et al. (2024)	CARS suicide reduction model reduced suicidal behaviour compared to TAU	*N* = 401	Study showed significant reductions in suicidal behaviours and admissions at 12 month follow up	Moderate certainty due to low risk of bias, high indirectness and low imprecision
Mitchell et al. (2020)	PAPU model compared across 3 hospitals and pre‐post	N/A	Study showed significant reductions in LOS at one hospital	Moderate to high certainty due to low risk of bias and inconsistency

Abbreviations: AOD, alcohol and other drug; CALM, crisis assessment link and management; CARS, programme for management of suicidal behaviours and suicide prevention; ED, emergency department; LOS, length of stay; PAPU, psychiatric assessment and planning unit.

## 3 Results

### 3.1 Initial Search Yield

**FIGURE 1:**
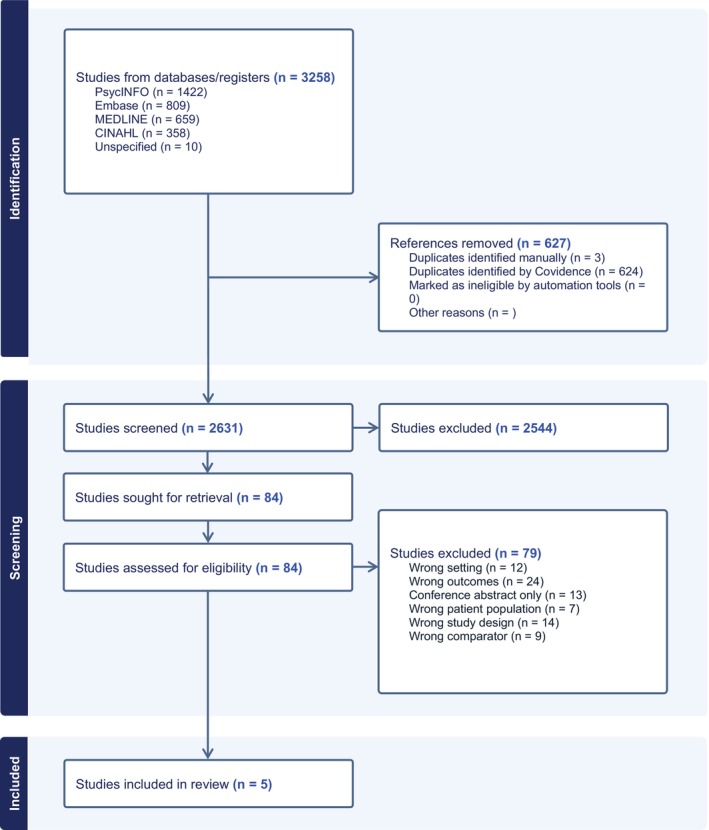
PRISMA flowchart of screening selection.

A total of 3258 references were identified in the original search, of which Covidence removed 627 duplicates, leaving 2631 to be screened by title and abstract. Of these, 84 were reviewed in full text and subsequently 79 articles were excluded (see Figure 1.) Specifically, we excluded 13 articles for being a conference abstract, 12 articles for wrong setting, 7 articles for wrong patient population, and 24 articles for wrong outcomes, which included articles that mentioned models of assessment but did not evaluate the model as one of their outcomes. Five articles were included in this study and narratively reviewed.

### 3.2 Description of Studies

An overview of the study characteristics included in this review is provided in Table 1. The total number of participants across all studies was *N* = 4578 with a mean age across studies of 39.1 years, and 52.9% participants were female. The de Santiago‐Díaz et al. [26] study included the emergency departments of University Hospital of Valdecilla in Cantabria, Spain. The study evaluated the outcomes of the Programme for Management of Suicidal Behaviours and Suicide Prevention (CARS), which compared the CARS programme to patients receiving treatment as usual (TAU) at 6 and 12 month follow up. The Howard et al. [27] study included a tertiary metropolitan hospital emergency department in Melbourne, Australia and evaluated an Alcohol and Other Drug (AOD) screening tool and brief intervention, the CARS model included a 32‐month pilot program. The Lester et al. [28] study included a metropolitan medical centre in Ohio, US and conducted a retrospective chart review looking at outcomes for patients included in the Crisis Assessment Link and Management (CALM) service and evaluated pre and post CALM implementation comparisons. The Browne et al. [6] paper included a metropolitan hospital in Melbourne, Australia and evaluated length of stay pre and post implementation of a Psychiatric Assessment and Planning Unit (PAPU) in the emergency department. The Mitchell et al. [29] study included three metropolitan hospitals in Melbourne, Australia, and evaluated the PAPU model during a 12‐month period and compared to a pre‐PAPU period, ED length of stay was measured and patient and staff interviews and surveys were analysed.

Two specialised models were identified in the search; de Santiago‐Diaz et al. [26] and Howard et al. [27] looked specifically at sub‐samples of the general mental health populations, focussing on suicidal behaviour or AOD. Although these are specialised MH models, they fit our inclusion criteria and therefore have been included in our review.

#### 3.2.1 Length of Stay Assessments

Three studies (Lester et al. [28], Browne et al. [6] and Mitchell et al. [29]) reported on length of stay before and after the model's intervention. Lester et al. [28] reported reduced length of stay using the CALM model in both ED and hospital stays, reporting a reduction from 9.5 to 7.3 h (24%) in ED and a reduction of 46.2 to 31.4 h (32%) in hospital stays after admission. Browne et al. [6] reported on ED length of stay before and after the implementation of a PAPU, results show a reduction in patients waiting over 24 h from twenty‐five to one from the first 12 months of opening the PAPU. Mitchell et al. [29] measured and reported on the length of stay between 3 metropolitan hospitals over a 12‐month period, The Austin, Peninsula Health and Eastern Health, who have implemented the PAPU model. The review also compared each hospital pre and post PAPU implementation. Results reported a decrease in LOS wait times pre‐post at The Austin, but an increase at Peninsula and Eastern Health sites, the authors state this could be due to a large increase in MH presentations from pre‐PAPU to 12 months post PAPU implementation. Additionally, The Austin had the shortest wait time in ED at 5 h compared to 6 h at Peninsula Health and Eastern Health.

#### 3.2.2 Specialised Models

The de Santiago‐Diaz et al. [26] study evaluated the effectiveness of the suicidal reduction CARS program compared to TAU and reported that suicidal behaviour had reduced by 35.5% at 12 months follow up in the CARS treatment group and reported reduced occurrence of hospital admissions. Howard et al. [27] evaluated an AOD screening and Brief Intervention model in ED and reported that over the 32‐month pilot, (*N* = 1100) patients underwent AOD assessment and 95% of those also received the subsequent Brief Intervention, resulting in 65% stating they would change their AOD habits in future.

#### 3.2.3 Short Stay Models

Three out of five articles included in this review measure and evaluate models that include short stay spaces within the ED or adjacent to the ED, across multiple hospitals in Melbourne Australia and Ohio, USA. These models focused on the entire MH cohort, whereas the other two articles only focussed on a specialised cohort. As evidence is scant surrounding MH models/assessments of care in ED, it is important to note that of the 5 included articles, 3 evaluated multiple bed‐based short stay models and reported positive outcomes. Utilising bed‐based short stay models such as the CALM model has shown to decrease hospital LOS, which in turn improved ED flowthrough and can provide relief to chaotic ED environments with high MH presentations. Likewise, the PAPU model reports qualitative results that reflected the PAPU to be a sanctuary or respite from the busy ED for MH patients.

### 3.3 Risk of Bias Assessment

Browne et al. [6] and Howard et al. [27] reported a total score of high risk of bias using the Revised version of the risk of bias tool for non‐randomised studies (RoBANS 2) [23] (see Table 2) both reported high risk on d4 ‘measurement’, d6 ‘outcome measure’ and the Browne et al. [6] article reported high risk on d7 ‘incomplete data and d8 selective reporting’. The data analysed from the Howard et al. [27], Lester et al. [28] and de Santiago‐Diaz et al. [26] articles all reported low risk of bias (see Table 2).

### 3.4 Certainty Assessment

Table 3 displays certainty of evidence assessment and is presented according to the GRADE guidelines for a narrative assessment [24, 25]. All studies reported moderate certainty of evidence except for the Mitchell et al. [29] article which reported moderate to high levels of certainty.

## 4 Discussion

We aimed to explore the current literature describing models of mental health assessments within emergency departments worldwide. This narrative review provides a detailed examination and builds upon previous reviews looking to identify similar models.^13,17,26^ Included in this review is 4 models across 5 articles, due to the health service based nature of emergency departments and assessment models, many models are not published in academic or medical literature as they are internal processes within each health service, this may attribute to a small number of models being found within this review.

Additionally, the emergency department is a challenging setting to make changes or trial system‐level interventions such as assessment models, largely due to the daily crises and chaotic environment for staff and patients. In order to make system‐level changes within an ED, a lot of factors need to be addressed; this is highlighted in a similar review by Johnson et al. [30], factors such as increases in funding, staff and available time to create and then embed a model of assessment [30].

Our results reported 4 models, with 2 of the models reflecting very similar practices, the Mitchell et al. [29] and Browne et al. [6] articles reported on PAPU models, which recommended a dedicated space with beds and staff in the ED for mental health patients, where Lester et al. [28] recommended the CALM model which also utilised beds and dedicated staff in ED. These models are robust and reported moderate certainty of evidence and with Lester et al. [28] reporting a low risk of bias and Browne et al. [6] reporting a high risk of bias, however, these models measured the variable ‘length of stay’ (LOS) in the ED and reported improvement pre‐post. Reporting on LOS in the ED as a primary variable limits the results as patients can be moved from the ED to others areas of the hospital which could incorrectly be reported as reduced length of stay, such as transporting patients to another waiting room to clear up space in the ED, which is recorded as the patient leaving ED. Future studies would benefit from including more variables in the analysis such as patient re‐presentation to ED for the same issue or qualitative data on patients experience in the ED and short stay unit.

### 4.1 Narrative Review of Included Studies

These studies report positive benefits of a model that includes short‐stay beds within the ED. As the ED is a crisis‐driven and chaotic environment [8, 9, 30, 31] it is important to consider the MH population that is seeking support there. A dedicated space and MH clinical staff in the ED to support and manage MH patients (as seen here within these models) seem to play an important role in the measure of length of stay within both ED and the hospital overall. Reducing the patients' length of stay, particularly in an MH context, can mitigate extra stressors that can be placed on the patients within the ED setting, such as increased exposure to traumatic and chaotic environments that can be detrimental to their health. Additionally, the Browne et al. [6] study reported a 50% reduction in the total number of patients being restrained in the ED and a reduction in Code Greys called to the ED. Creating an environment which has a dedicated location for MH patients shows a decrease in stressors and trauma on the patients, whether this is through a PAPU model or a similar model such as the CALM model Lester et al. [28].

Mitchell et al. [29] conducted a mixed methods evaluation of the PAPU model across 3 metropolitan hospitals in Melbourne Australia, they reported the PAPU was well accepted by patients and feedback was overwhelmingly positive. This study also reported that the PAPU models improved the safety of both staff and patients. The diagnosis of patients that presented to the PAPUs in this study varied and was inclusive of MH needs, including AOD patients. However, the Mitchell et al. [29] study reported in one hospital (The Austin) there was a decrease in LOS but at the other 2 hospitals there was an increase in ED LOS, this could have been due to the increased MH presentations at the 2 hospitals compared to The Austin, this could have allowed for more patients to be admitted into the PAPU therefore reducing ED LOS. Location of the hospital and PAPU or short‐stay unit is an important variable to consider when planning to make changes to the ED and system. Mitchell et al., reports that the changes in ED LOS (both increased and decreased) and improvements may be due to other factors such as increased ED system governance, better ED liaison or the increase in MH presentation. Specifically, there was a 41.5% increase in MH presentations over the 3 years of the evaluation across all 3 hospitals, this is something to consider when designing and implementing a new short stay unit in any ED.

An important theme that is highlighted here is the use of short stay beds and a MH space within the ED, with the increasing number of MH patients presenting to the ED, designing and embedding a model such as CALM or a PAPU has shown to provide better and faster support for those seeking MH care. Despite the increased numbers of AOD presentations to the ED and the success of the Howard et al. [27] AOD brief intervention model, this model does not address MH needs specifically and therefore leaves out a large number of patients. Similarly, the de Santiago‐Diaz et al. [26] CARS suicidality model only caters to patients whose symptoms involve suicidality, limiting patients' access. The specialised models also require active follow up and group attendance; this is not feasible in a rural and regional setting where some patients are travelling up to 4 h to the ED to seek MH supports, as such with regional Victoria. This narrative review reports that a possible solution to increased numbers of MH patients in ED is to create a physical space, with MH dedicated staff and some short stay beds to cater to the MH patients' needs. Taking into consideration the organisational and structural issues that may arise with trying to implement a model such as PAPU or CALM, this review adds to the evidence that short stay models are successful over time in multiple hospitals, and this review could be considered when making policy decisions, particularly in an Australian context.

### 4.2 Quality, Completeness and Generalisability of Evidence

This review aimed to identify models of mental health assessment in emergency departments; we identified 5 models, of which two reported high risk of bias (Browne et al. [6] and Howard et al. [27]) and all articles reported moderate certainty of evidence. Specifically, de Santiago‐Diaz et al. [26] and Mitchell et al. [29] both reported low risk of bias, but de Santiago‐Diaz et al. [26] scored high on indirectness and Mitchell et al. [29] scored low on inconsistency.

The 5 included articles in this review reported numerous and differing outcome measures, therefore making it challenging to form comparisons, for example the Mitchell et al. [29] article had 4 primary outcomes which were reported across 3 hospital settings using the model.

Three of the articles measured LOS as their primary outcome, Mitchell et al. [29], Browne et al. [6] and Lester et al. [28], although this measure does provide information on adequate treatment times, it is not a direct measure of patient outcomes, symptom improvement, or patient satisfaction with their treatment, this limits the completeness domain in the GRADE quality of evidence. Lastly, the practicality of implementing these models is directly connected with funding and possible rebuilds of the entire emergency department, this may not be feasible in many health services and limits the overall generalisability of these models.

### 4.3 Limitations

This narrative review is limited by the lack of research published surrounding mental health models of assessment in emergency departments; this could be due to internal health service models not being assessed, researched and published within the literature base. Despite using broad search terms and multiple databases, there could be language differences when searching for “models” or “assessment” which may have limited our search strategy. Lastly, three out of the five models reviewed were Australian metropolitan based, two of them assessing the same PAPU model which creates a bias in the results reported within this narrative review.

### 4.4 Clinical Implications

The inclusion of a mental health assessment model that requires the emergency department to have either a physical space for mental health patients, and/or dedicated mental health staff has shown to be partially successful in emergency departments, across the 5 articles in this narrative review. The articles collectively report on the possibility to reduce ED overcrowding and the clinical burden of ED staff, as highlighted by similar reviews of narrative mental health care in emergency departments [17, 30].

## 5 Conclusion

To conclude, the short stay bed‐based models such as the CALM model and the PAPU model have displayed successful outcomes. The PAPU model consistently reports reduced ED‐LOS when comparing pre‐PAPU implementation to 12 months post‐PAPU, as reported in 2 articles, and also reports good acceptability of the model from staff and patients. The CALM model also displayed positive results with its bed‐based model which reported reduced ED‐LOS and hospital LOS and improved patient care. The literature suggests that regardless of the type of model used, they all require dedicated ED mental health staff, and a physical area located in the ED for mental health staff and patients to be seen, with the addition of short stay beds. Considering the increase in mental health presentations to ED's, future directions include hospitals providing trained mental health ED staff and or providing mental health beds in the ED, as a way to possibly alleviate the pressures on ED staff and provide better supports for patients.

## Funding

The authors have nothing to report.

## Conflicts of Interest

The authors declare no conflicts of interest.

## Supporting information


**Data S1:** emm70268‐sup‐0001‐Supinfo.pdf.

## Data Availability

Data sharing not applicable to this article as no datasets were generated or analysed during the current study.

## Appendix 1 Search Strategy for Medline Complete

“assessment” or assessment model or screen or model of care.

“emergency room” or accident and emergency, or emergency department or ED or ER.

“mental health” or mental disorder or psychiatric disorder or mental illness or mental disorder or psychiatric illness.
